# Effect of No Tillage System on Soil Fungal Community Structure of Cropland in Mollisol: A Case Study

**DOI:** 10.3389/fmicb.2022.847691

**Published:** 2022-06-16

**Authors:** Ming Gao, Haiyu Li, Meng Li

**Affiliations:** Northeast Institute of Geography and Agroecology, Chinese Academy of Sciences, Harbin, China

**Keywords:** conservation tillage, fungi, diversity, pathogens, SEM, soil organic carbon

## Abstract

Conservation tillage is generally regarded as a sustainable farming system for the future. The fungal community structure has a strong response to conservation tillage. However, how the conservation tillage system affects the soil fungal community structure is little known. Using the high-throughput sequencing technology, the soil fungal community was explored under no tillage (NT) and conventional tillage (CT) in Northeast China Mollisol. The copy number of fungal genes in NT20 was significantly lower than that in other treatments. NT changed the composition of soil fungal communities at the taxonomic level of phylum and genus. The diversity indices of the soil fungal community in no tillage at soil depths of 0–5 cm (NT5) were significantly higher than those in soil depths of 5–20 cm (NT20). The fungal community under NT and CT could form a good cluster distribution and NT5, conventional tillage at soil depths of 0–5 cm (CT5) and 5–20 cm (CT20) had specific indicator species. Most of the potential pathogens were significantly higher in NT5 than in NT20. Tillage and soil depth could explain 64% of the diversity and 95% of the composition of the fungal community, which indirectly changed the diversity and composition of fungi by using soil organic carbon, pH value, and soil bulk density. Furthermore, soil organic carbon (SOC) best explained the soil fungal community, followed by soil pH. The study indicated that the NT system had a comprehensive effect on the soil fungal community and SOC is the most crucial factor in determining this community.

## Introduction

Traditional tillage has a certain effect on soil’s physical, chemical, and biological properties, thus affecting soil productivity. In the seedling stage of crops, traditional farming could lead to farmland soil erosion and pose a certain threat to the subsequent agricultural production capacity. Studies have shown that conservation tillage could improve crop water use efficiency and nutrient levels to improve crop yield. Thus, it is a sustainable farmland management measure (Hobbs et al., 2008; Zhang et al., 2011).

The soil fungal community plays a crucial role in the biochemical cycle, organic matter transformation, and disease development and control (Jirout et al., 2011; Latz et al., 2021). The diversity of the soil fungal community is affected by many factors, such as soil type, physical, chemical, and geographical properties (Tkacz et al., 2015). Tillage changes soil’s physical and chemical properties, thus, affecting the fungal community structure (Jirout et al., 2011). Therefore, the fungal community structure has a strong response to conservation tillage and it is a powerful tool to monitor the environmental change (Arenz and Blanchette, 2011; Nie et al., 2012). The community classification structure of soil fungi is related to functional diversity. The functional diversity of soil fungi can improve plant nutrient absorption, prevent crop diseases, and directly affect crop yield (Deacon et al., 2006; Heijden et al., 2010). Soil-borne plant pathogenic fungi seriously affect the function of the agricultural ecosystem (Corredor-Moreno and Saunders, 2020). Owing to the interaction between soil-borne plant diseases and soil microorganisms, assessing the soil microbial community structure, which aims to suppress plant pathogens, is essential (Mazzola, 2004). Fungal diversity has a positive effect on soil-borne plant pathogen suppression and productivity (Wehner et al., 2010). Some studies have shown that tillage could affect the diversity of soil saprophytic fungal communities due to the physical destruction of mycelium (Kihara et al., 2012; Kabiri et al., 2016). Up to now, how tillage affects the soil fungal pathogen populations remains largely unknown.

Many studies have shown that the fungal community structure is affected by conservation tillage (Schlatter et al., 2017; Sun et al., 2018; Zhang et al., 2021). However, a meta-analysis conducted by Li et al. in 2020 showed that tillage has no consistent effects on fungi (Li et al., 2020) because local conditions, such as soil type, climatic conditions, and abiotic factors, play an important role in shaping the fungal community in arable soil (Tedersoo et al., 2014). Mollisol in Northeast China is famous for its high organic matter content and high crop productivity. Some studies have shown that the topsoil of Mollisol has been seriously eroded and the potential of soil productivity has declined (Liu et al., 2010). Conservation tillage could prevent soil erosion and improve the soil physical and chemical properties of cropland in Northeast China (Liang et al., 2009). However, the structure of the soil fungal community under no tillage (NT) with residue retention in Northeast China is still unclear. Therefore, the present study, based on the long-term experiment, aimed to explore the changes in the fungal community under NT and conventional tillage (CT) and verify the relationship between soil fungal community and soil physicochemical properties.

## Materials and Methods

### Site Description

This study is based on the farmland ecosystem in Hailun City, Heilongjiang Province (N 47°26′, E 126°38′). The Hailun station was founded in 1978 and it became the national field scientific observation station in 2005. It is located in the central area of the Mollisol belt in Northeast China. Being 240 m above sea level, it is located in the temperate continental monsoon climate zone, with high temperature and rainy in summer, cold temperature and dry in winter, annual average temperature of 1.5–2.9°C, annual average rainfall of 500–600 mm, annual average effective accumulated temperature (≥ 10°C) of 2400–2500°C, annual average sunshine duration of 2,600–2,800 h and frost-free period of 120–130 days.

### Experimental Design

The research experiment, including NT and CT, was set up in 2004 in the Hailun Station. Each tillage system had three replicates, which were used for the complete randomized block design. Maize (*Zea mays*) and soybean (*Glycine max*) rotations were adopted. The area of each plot was split from other plots by using a 0.7 m-wide barrier. Each plot was 8.4 m × 40 m. NT only harvested crop seeds. Except for the harvested seeds, all other biomasses covered the whole plot surface evenly. At the beginning of May every year, crops (corn or soybean) were sown with no till planters and the soil was kept as it was. In CT, all seeds and residues were removed after harvest in October and then rotary tillage was used, with a depth of 20 cm and a ridging height of 15 cm. In early May of next year, the soil was planted with a traditional planter. After planting, it was ridged twice every 15 days. Except for farming operations, NT and other management of CT farming were the same. In addition, 138 kg ha^–1^ nitrogen, 51.75 kg ha^–1^ phosphorus (P_2_O_5_), and 15 kg ha^–1^ potassium (K_2_O) were applied to maize, and 20.25 kg ha^–1^ nitrogen, 51.75 kg ha^–1^ phosphorus (P_2_O_5_), and 15 kg ha^–1^ potassium (K_2_O) were applied to soybean.

### Sample Collection and Analysis

In late July 2018, soil samples of NT and CT were collected in the crop growing season. The crops in that season are soybeans. The soil was sampled randomly with two-sampling depths of 0–5 and 5–20 cm. In each treatment, six samples were collected. The six samples were evenly mixed as a soil sample. Each treatment was repeated three times, and a total of 12 composite samples were collected. The soil samples were stored in a sterile sealed bag with ice cubes and brought back to the laboratory as soon as possible. Plant residues, stones, and other impurities were removed from the fresh soil within 1 day and then passed through a 2 mm sieve and mixed evenly. Subsequently, each sample was divided into two parts. The subsamples were stored at –20°C for analyzing the microbial community and the remaining were dried for determination of soil physicochemical properties.

Soil bulk density (BD) was measured from oven-dried undisturbed cores as mass per volume of oven-dried soil, with three replicates. Soil pH was determined in a 1:2.5 soil/water suspension and deionized water with a pH meter. Soil organic carbon (SOC) concentration was measured by the wet oxidation process with dichromate in accordance with the Walkley–Black method. Total nitrogen (TN) was measured using the Kjeldahl method. Soil N availability (NO_3_^–^–N and NH_4_^+^–N) was determined by AA3 continuous-flow analyzers (AA3, Seal Analytical, Germany).

### Soil DNA Extraction and Quantitative Real-Time PCR

In accordance with the manufacturer’s directions, the total soil DNA was extracted from 0.5 g of soil sample with FastDNA spin kits for soil (MOBIO PowerSoil DNA Isolation Kit). The NanoDrop spectrophotometer (Thermo Scientific, Wilmington, United States) was used to measure the DNA quantity and quality. All the extracted DNAs from soil were stored in –20°C refrigerator for further analysis.

The fungal community abundance was explored by quantitative PCR using primers for specific genes or genetic regions, which was investigated by targeting the ITS region with the primer set ITS1F/ITS2R: (50-ACTTGGTCATTTAGAG-GAAGTAA-30) and ITS2 (50-BGCTGCGTTCTTCATCGA TGC-30; Mukherjee et al., 2014). Amplification was performed under the following conditions: denaturation for 10 min at 95°C, 40 cycles of amplification for 15 s at 95°C and 50 s at 60°C, followed by a final melt curve of 15 s at 95°C, 1 min at 60°C with an increase of 0.3°C to a final temperature of 95°C. The 25 μl reaction mixture contained the following: 15 μl of 1 × SYBR Premix Ex Taq, 0.25 μl of each primer, 4 μl of dNTPs (2.5 mmol L^–1^), and 1 μl of DNA template DNA (50 ng). Negative controls contain 2.0 μl of double distilled water. The PCR products were electrophoresed using a 2.0% agarose gel, with the ITS fragment exhibiting a size close to that expected. Other details can be seen in the article by Liu et al. (2015).

### Illumina MiSeq Sequencing and Processing of Sequencing Data

The triplicate amplicons of each sample were combined, purified, quantified, and then sequenced using a MiSeq PE300 instrument obtained from the platform at Majorbio Bio-Tec Co., Ltd. (Shanghai, China). Quantitative insights into microbial ecology (QIIME) version 1.9.1 was used to obtain raw sequences (Caporaso et al., 2010). The sequence reads were assigned to each sample in accordance with the unique barcode, after which the barcodes and primers were removed. The paired reads were joined with FLASH (version 1.2.7). The trimmed sequences were removed by the UCHIME algorithm (Edgar et al., 2011). UPARSE was used to cluster the remaining sample reads into operational taxonomic units at a 97% similarity level (Edgar, 2010).

### Statistical Analysis

One-way ANOVA was used to analyses significant differences, and SPSS 18.0 was used to process the data. Fungal community diversity was determined using the Chao 1 and Shannon indices. Non-metric multi-dimensional scaling (NMDS) was used to analyses the fungal communities’ structure (Lozupone et al., 2011). The difference in each treatment was identified using ANOSIM similarity analysis. Indicator species were analyzed with the indicator species data package of R software (version 3.5.0) and the species with *p* < 0.05 were selected as the indicator species (Dufrêne and Legendre, 1997). Potential pathogens and beneficial fungi were analyzed through Funguild (Nguyen et al., 2016). Then, in accordance with the published literature, changes in potential pathogenic and beneficial fungi were identified. The relationship between soil physicochemical properties and fungal diversity was determined using the Pearson correlation coefficient. The introduction of structural equation modelling (SEM) was given by Li et al. (2021).

## Results

### Effect of NT on Soil Fungal Abundance and Taxonomic Classification

Under NT and CT treatments, the gene copy number of NT20 was significantly lower than that of NT5, CT5, and CT20 (Table 1). In this experiment, 780,697 optimized sequences were obtained from all samples, and 42,958–72,002 optimized sequences (average of 65,058 optimized sequences) were obtained from each sample. The average length of the optimized sequences was 235 bp, with a length of 230–246 bp (Supplementary Table 1). Based on a 97% similarity level, seven phyla, 24 classes, 76 orders, 157 families, 311 genera, and 1,348 OTUs were identified. The total number of OTUs in NT5 treatment was significantly higher than that in NT20, CT5, and CT20. The soil fungal data set has been saved in NCBI with accession number PRJNA778460.

**TABLE 1:** Soil fungal gene abundance and diversity indices at different soil depths under NT and CT.

	ITS copies (× 10^6^)	Chao 1	Shannon
NT5	12.84 ± 1.54a	751.40 ± 63.38a	4.23 ± 0.12a
NT20	3.63 ± 1.18b	484.99 ± 60.17b	3.40 ± 0.34b
CT5	12.03 ± 5.43a	536.59 ± 67.53b	3.40 ± 0.72b
CT20	10.42 ± 2.62a	541.78 ± 20.51b	3.67 ± 0.10ab

*NT5, no tillage at soil depths of 0–5 cm; NT20, no tillage at soil depths of 5–20 cm; CT5, conventional tillage at soil depths of 0–5 cm; CT20, conventional tillage at soil depths of 5–20 cm. Different small letters indicated difference between different treatments (P < 0.05).*

**FIGURE 1:**
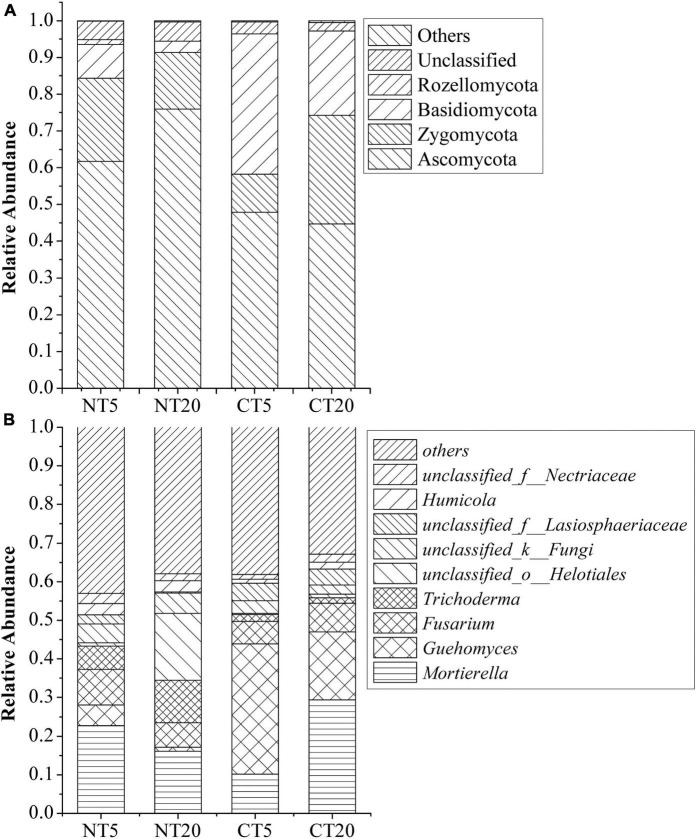
Relative abundance of predominant fungal at phylum **(A)** and genus **(B)** levels under NT and CT.

The dominant phyla of fungi in NT and CT included Ascomycota (44.71–75.93%), Zygomycota (10.38–29.50%), Basidiomycota (3.03–38.16%), Rozellomycota (0–1.33%), Unclassified (2.30–5.25%), and others (0.17–0.51%), as shown in Figure 1A. The relative abundance of Ascomycota was the highest. The phyla with significant changes between NT and CT included Ascomycota, Zygomycota, and Basidiomycota.

The dominant genera of fungi in NT and CT were *Mortierella* (10.16–29.35%), *Guehomyces* (1.02–33.7%), *Fusarium* (5.83–9.19%) *Trichoderma* (1.4–11.03%), *unclassified_o__Helotiales* (0.29–17.3%), *unclassified_k__Fungi* (2.33–5.31%), *unclassified_f__Lasiosphaeriaceae* (0.27–4.52%), *Humicola* (0.98–2.98%), *unclassified_f__Nectriaceae* (1.22–2.62%), and others (32.84–43.06%), as shown in Figure 1B. The genera with significant changes between NT and CT included *Mortierella*, *Guehomyces*, *unclassified_k__Fungi*, *unclassified_f__Lasiosphaeriaceae*, and *tetracladium*.

### Effect of No Tillage on Soil Fungal Community Diversity and Composition

**FIGURE 2:**
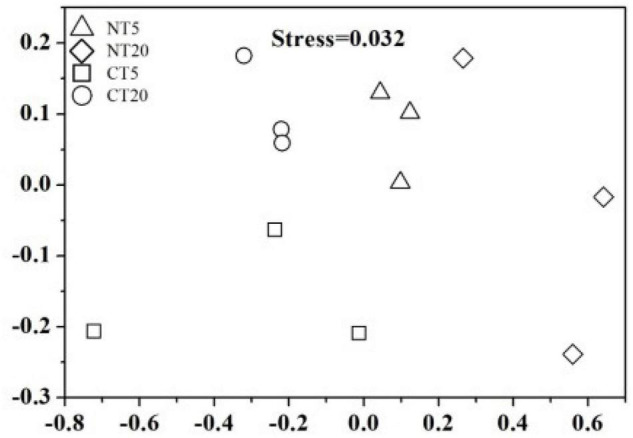
NMDS ordinations of soil fungal communities under NT and CT.

The Chao 1 and Shannon indices at soil depths of 0–5 cm were significantly higher than those at 5–20 cm under NT, while no significant difference was found between 0 and 5 cm soil depths and 5–20 cm soil depths under CT (Table 1). The NMDS analysis indicted that each treatment of the fungal communities forms a good cluster distribution (Figure 2).

### Effect of No Tillage on Soil Fungal Community Indicator Species

The indicator species of NT5 were OTU703, OTU1288, OTU422, OTU1325, OTU818, OTU329, and OTU538, which may be *Coniochaetales_sp*, *unclassified_k__Fungi*, *Cordyceps_bassiana*, *unclassified_f__Plectosphaerellaceae*, *unclassified_k__Fungi*, *unclassified_g__Fusarium* and *unclassified_k__Fungi*, respectively (Table 2); the indicator species of CT5 were OTU115 and OTU867, which may be *comparison_k__Fungi* and *Cephalotheca_sulfurea*, respectively. The indicator species of CT20 was OTU 840, which may be *Lasiosphaeriaceae_sp*.

**TABLE 2:** Indicator species of fungal community under NT and CT.

Treatment	Indicator species	Stat	*P*	Taxonomy
NT5	OTU703	1	0.014	*s__Coniochaetales_sp*
NT5	OTU1288	1	0.014	*s__unclassified_k__Fungi*
NT5	OTU422	0.987	0.014	*s__Cordyceps_bassiana*
NT5	OTU1325	0.986	0.014	*s__unclassified_f__ Plectosphaerellaceae*
NT5	OTU818	0.985	0.014	*s__unclassified_k__Fungi*
NT5	OTU329	0.984	0.014	*s__unclassified_g__Fusarium*
NT5	OTU538	0.974	0.014	*s__unclassified_k__Fungi*
CT5	OTU115	1	0.017	*s__unclassified_k__Fungi*
CT5	OTU867	0.977	0.017	*s__Cephalotheca_sulfurea*
CT20	OTU840	0.976	0.012	*s__Lasiosphaeriaceae_sp*

*Stat is the average of specificity and fidelity. If specificity = 1, calpurp occurs only in that group; if fidelity = 1, all sites within the group contain calpurp.*

### Effect of No Tillage on Soil Potential Pathogenic and Beneficial Fungi

**FIGURE 3:**
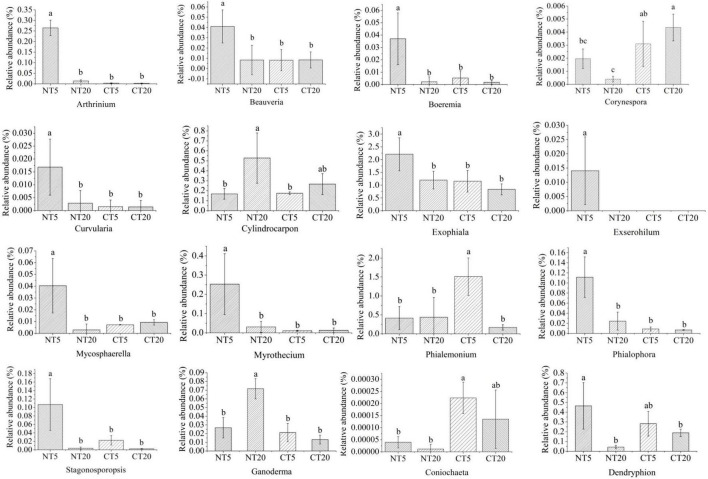
Relative abundance of soil potential pathogens fungi under NT and CT. Different small letters indicated difference between different treatments (*P* < 0.05).

**FIGURE 4:**
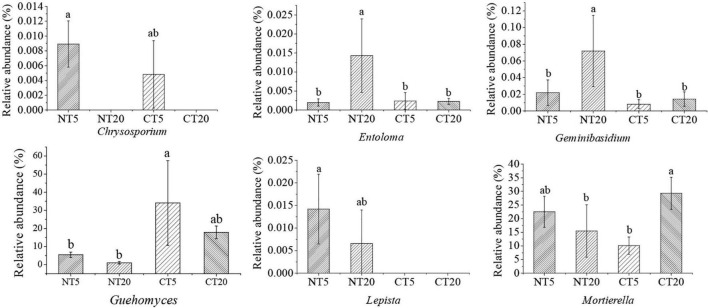
Relative abundance of soil potential beneficial fungi under NT and CT. Different small letters indicated difference between different treatments (*P* < 0.05).

The genera with significant changes in the relative abundance between NT and CT were identified, and then 16 kinds of potential pathogenic fungi and six kinds of potential beneficial fungi were screened through Funguild and literature analysis. Except for *Cylindrocarpon*, *Phialemonium*, and *Coniochaeta*, the other potential pathogen fungi were significantly higher in NT5 than in NT20, and no significant difference was observed between CT5 and CT20 (Figure 3). Compared with pathogenic fungi, no uniform trend was found in the community structure of potential beneficial fungi among different treatments. The *Chrysosporium* in NT5 was significantly higher than that in NT20 and *Entoloma* and *Geminibasidium* were significantly lower in NT5 than in NT20. Except for *Chrysosporium* and *Mortierella*, no significant difference was observed in the other potential beneficial fungi between CT5 and CT20 (Figure 4).

### Relationship Between Soil Fungal Community and Soil Physicochemical Properties

Correlation analysis of the diversity of the fungal community and soil physicochemical properties showed that BD had a significant correlation with fungal gene copies and NMDS 1; pH had a significant correlation with gene copies, Chao 1 and NMDS 1; TN had a significant correlation with Chao 1; and SOC had a significant correlation with Chao 1 and Shannon indices (Table 3).

**TABLE 3:** Correlation between soil fungal community and physicochemical properties (**p < 0.01; *p < 0.05).

	BD	pH	NO_3_^–^–N	NH_4_^+^–N	TN	SOC
ITS copies	−0.628*	0.777**	0.462	0.312	0.238	0.181
Chao 1	–0.427	0.707*	0.707	0.832	0.773*	0.768**
Shannon	–0.178	0.576	0.649	0.636	0.642	0.663*
NMDS 1	0.718**	−0.51*	−0.018	0.292	0.232	0.255
NMDS 2	0.021	0.34	−0.033	0.201	0.269	0.341

**FIGURE 5:**
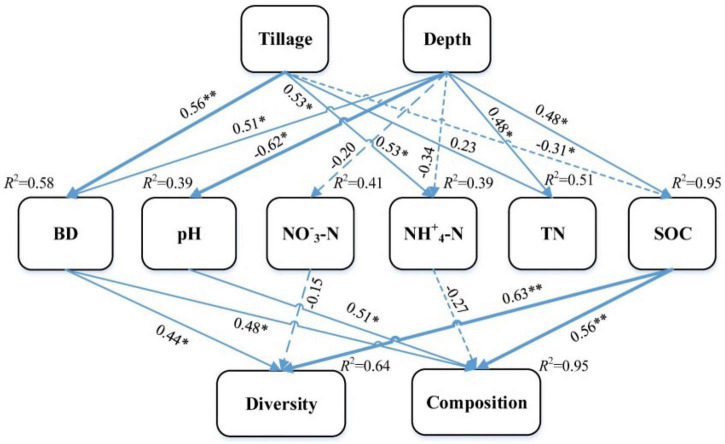
Structure equation models examining the effects of tillage and depth on soil fungal communities. SOC, soil organic carbon; TN, total nitrogen, NO^–^_3_–N and NH^+^_4_–N, soil N availability; BD, soil bulk density. A dashed line is a negative relationship and a solid line shows a positive relationship. Numbers above the arrows are standardized path coefficients. The arrow’s width is proportional to the strength of the relationship (^**^p < 0.01; *p < 0.05).

The effects of tillage and soil depth on soil fungal communities were predicted by SEM, and the final fitting SEM was good (χ^2^ = 53.35, df = 20, *p* < 0.01, RMSEA = 0.39, GFI = 0.55, AIC = 103.35). The results showed that tillage and soil depth could explain 64% of the diversity and 95% of the composition of fungal community (Figure 5). Tillage and soil depth had no direct effect on the fungal community but had a significant indirect effect. SOC affected the diversity and composition of the fungal community; pH affected the composition of the fungal community and BD affected the diversity and composition of the fungal community. In addition, SOC best explained the soil fungal community, followed by pH.

## Discussion

### Effects of No Tillage on Soil Fungal Community

In this study, Ascomycota had the highest relative abundance under NT and CT (Figure 1A). Ascomycota has been proven to be able to utilize the carbon released by roots (Hannula et al., 2012). The relative abundance of Ascomycota is significantly different between 0 and 5 and 5–20 cm soil depths under NT. This finding may be explained by NT5 having a large amount of residue. The Chao 1 index of the NT5 treatment was significantly higher than that of the NT20, CT5, and CT20 treatments (Table 1), indicating that NT resulted in higher fungal diversity in topsoil. The NT maintained higher ecosystem stability by reducing soil disturbance and increasing crop residue coverage (McNaughton, 1994). Therefore, the vertical change of the fungal community in the 0–20 cm tillage layer under NT should not be ignored. Other studies also corroborated that soil depth is a decisive factor in the diversity and composition of the fungal community under NT and similar conclusions have been drawn in forests and grasslands (Baldrian et al., 2012; Toju et al., 2016).

Indicator species are those whose biological or ecological characteristics could characterize the status of other species or the environment. The changes at the level of the whole community or ecosystem could be reflected by the existence of indicator species. The results of NT and CT indicated that OTU703 had the highest stat value of NT5 and OTU703 may be *Coniochaetales* (Table 2). Some studies indicate that *Coniochaetales* is related to wood erosion and soft rot (Stirling et al., 2017), consistent with the high residue of straw and high content of humus in NT5 of the present study.

The results of this study showed that most of the potential pathogenic fungi at 0–5 cm soil depths were significantly higher than those at 5–20 cm soil depths under NT; meanwhile, no significant difference was observed between 0 and 5 cm and 5–20 cm soil depths under CT (Figure 3), possibly because NT5 had high organic matter content and was undisturbed, thus having a strong selectivity for the fungal community (Jha, 2010). Boyette et al. indicated that soybean straw returned provided conditions for the survival of pathogenic fungi and provided the main inoculants for some diseases (Boyette et al., 2020). Soil borne pathogens, such as *Fusarium*, survived and grew better in straw buried in soil. Storey et al. also showed that most of the pathogenic fungi existed in the 0–5 cm profile in NT sandy loam soil, similar to the results of this study (Storey et al., 2006). Many pathogens in the present study have been proven to have different functions. For example, *Beauveria* is a well-known pathogen that is distributed all over the world and has been used for biological control of pests (Zimmermann, 2007). *Arthrinium* is widely distributed in living and decaying plants, and 70 species were identified in *Arthrinium* (Wang et al., 2018). Some scholars believe that *Arthrinium* is the pathogen of wheat seedling fusarium wilt (Mavragani et al., 2007). *Boeremia* is thought to cause soybean black spot in Australia (Jones et al., 2011). *Corynespora* could cause cucumber brown spot (Wang et al., 2019). *Exophiala* is associated with the soybean cyst nematode (Hu et al., 2017). *Mycosphaerella* is the pathogen of plant leaf disease (Bailey et al., 2001). Myrothecium could cause leaf spot of gourd (Bhuiyan et al., 2016). *Stagonosporopsis* could cause calabash vine blight and pyrethrum wilt by affecting the seed germination process (Stewart et al., 2015). Compared with the potential pathogenic fungi, no consistent trend was identified in the community structure of the potential beneficial fungi among the treatments (Figure 4). *Chrysosporium* at 0–5 cm soil depths was significantly higher than that at 5–20 cm soil depths under NT and *Entoloma* and *Geminibasidium* at 0–5 cm soil depths was significantly lower than that at 5–20 cm soil depths under NT. Some studies have shown that *Chrysosporium* has potential application value in the bioremediation of dye-contaminated soil and it may have a certain decontamination effect on contaminated sites (Sa et al., 2018; Spadaro et al., 2020). *Geminibasidium* is an indicator species of soil fungal community change under acid rain stress (Nan et al., 2020). *Guehomyces* was the dominant fungi in the surface soil of straw returning (Sa et al., 2018).

### Relationship Between Soil Fungal Community and Physicochemical Properties

The SEM results showed that tillage and depth had no direct effect on the soil fungal community but had an indirect effect on the soil fungal community *via* soil physicochemical properties (Figure 5). This result confirmed the importance of soil properties in affecting the soil fungal community in the NT system. Previous studies have also concluded that the diversity of soil microorganisms has a certain correlation with soil physicochemical properties (Zhu et al., 2012). In the present research, tillage and soil depth could explain 64% of the diversity and 95% of the composition of fungal community. Soil depth had a greater influence than tillage on fungal community. It may be because the residue returning amount distribution is different in the soil depth profile of NT, which indicates “niche-based” mechanisms regulating fungal community assembly (Tokeshi, 1990; Sun et al., 2018). The results of SEM showed that SOC best explained the soil fungal community, followed by pH (Figure 5), suggesting that SOC is the most crucial factor in determining the fungal communities in Mollisol. Therefore, SOC and soil pH were the primary factors in explaining soil fungal and bacterial communities, respectively, in the NT system of Northeast China (Li et al., 2021).

## Conclusion

This study showed that long-term conservation tillage increased the diversity of soil fungal communities and potential pathogens in NT5 treatment, and shaped specific indicator species. The heterogeneity in the 0–20 cm soil depth of the no tillage system cannot be ignored. Tillage and soil depth change the diversity and composition of soil fungi by affecting SOC, pH, and BD. SOC is the preferred factor to explain the soil fungal community rather than pH.

## Data Availability Statement

The datasets presented in this study can be found in online repositories. The names of the repository/repositories and accession number(s) can be found in the article/Supplementary Material.

## Author Contributions

ML designed the manuscript. MG and HL participated in the writing of the manuscript. All authors contributed to the article and approved the submitted version.

## Conflict of Interest

The authors declare that the research was conducted in the absence of any commercial or financial relationships that could be construed as a potential conflict of interest.

## Publisher’s Note

All claims expressed in this article are solely those of the authors and do not necessarily represent those of their affiliated organizations, or those of the publisher, the editors and the reviewers. Any product that may be evaluated in this article, or claim that may be made by its manufacturer, is not guaranteed or endorsed by the publisher.
