# Supplementary material for: Effect of No Tillage System on Soil Fungal Community Structure of Cropland in Mollisol: A Case Study
Source: Front Microbiol. 2022 Jun 16;13:847691. doi: 10.3389/fmicb.2022.847691 (PMC9244396; doi:10.3389/fmicb.2022.847691)
Supplement: Supplementary file 1 [file Data_Sheet_1.docx]

**Supplementary section:**

**Table S1** MiSeq-sequenced fungal data based on ITS rRNA gene

| Sample\Info | Seq_num | Base_num | Mean_length | Min_length | Max_length |
| --- | --- | --- | --- | --- | --- |
| NT5_1 | 68311 | 15986998 | 234 | 160 | 440 |
| NT5_2 | 66003 | 15506761 | 235 | 160 | 433 |
| NT5_3 | 72002 | 16892656 | 235 | 160 | 438 |
| NT20_1 | 57174 | 13699858 | 240 | 161 | 433 |
| NT20_2 | 59457 | 14651942 | 246 | 161 | 433 |
| NT20_3 | 69245 | 16115974 | 233 | 164 | 446 |
| CT5_1 | 70073 | 16726883 | 239 | 161 | 414 |
| CT5_2 | 70835 | 16558355 | 234 | 161 | 437 |
| CT5_3 | 66989 | 15390940 | 230 | 175 | 424 |
| CT20_1 | 67590 | 15858750 | 235 | 161 | 417 |
| CT20_2 | 42958 | 9911197 | 231 | 167 | 428 |
| CT20_3 | 70060 | 16237450 | 232 | 161 | 417 |


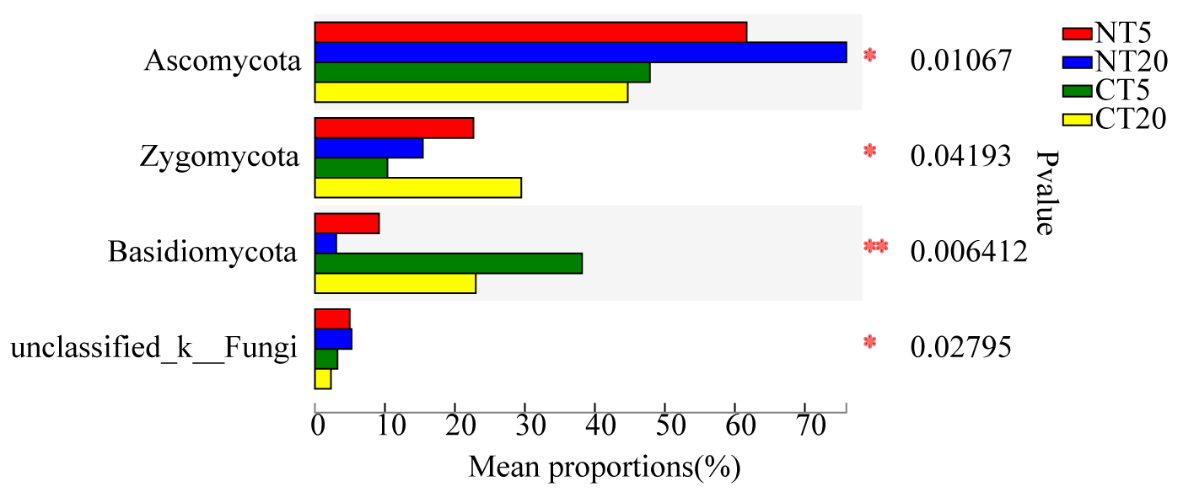


**Figure S1.** Difference in soil fungal community at the phylum level under NT and CT.


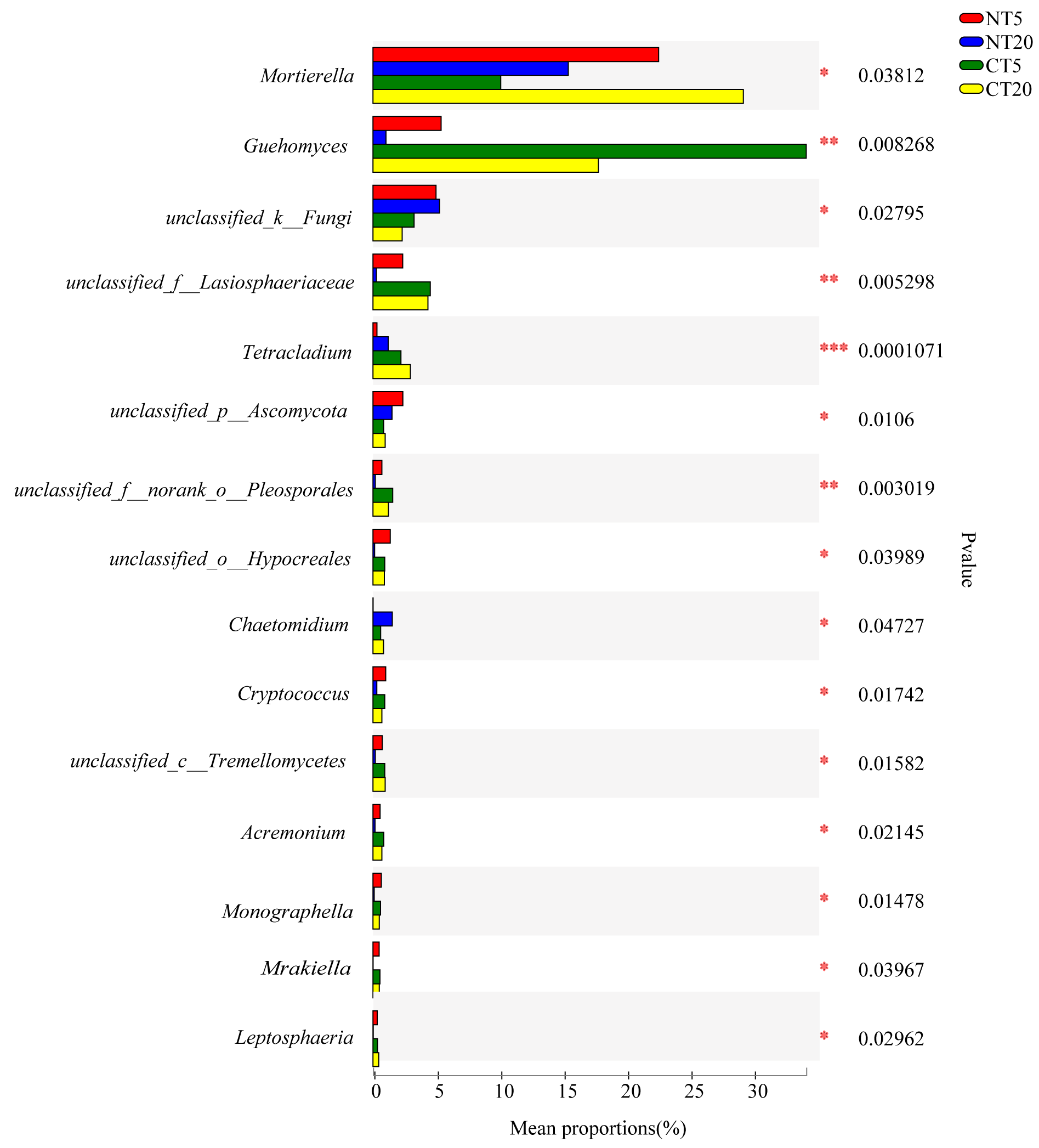


**Figure S2.** Difference in soil fungal community at the genus level under NT and CT.
